# A Population-Based Study of Childhood Cancer Survivors' Body Mass Index

**DOI:** 10.1155/2014/531958

**Published:** 2014-01-09

**Authors:** Echo L. Warner, Mark Fluchel, Jennifer Wright, Carol Sweeney, Kenneth M. Boucher, Alison Fraser, Ken R. Smith, Antoinette M. Stroup, Anita Y. Kinney, Anne C. Kirchhoff

**Affiliations:** ^1^Cancer Control and Population Sciences Research Program, Huntsman Cancer Institute, 2000 Circle of Hope, Salt Lake City, UT 84112, USA; ^2^Department of Pediatrics, University of Utah School of Medicine, 30 N. 1900 E, Salt Lake City, UT 84132, USA; ^3^Center for Children's Cancer Research, Huntsman Cancer Institute, 2000 Circle of Hope, Salt Lake City, UT 84112, USA; ^4^Department of Internal Medicine, Division of Epidemiology, University of Utah, 295 Chipeta Way, Salt Lake City, UT 84132, USA; ^5^Department of Oncological Sciences, Huntsman Cancer Institute, 2000 Circle of Hope, Salt Lake City, UT 84112, USA; ^6^Pedigree and Population Resource (Utah Population Database), Huntsman Cancer Institute, 2000 Circle of Hope, Salt Lake City, UT 84112, USA; ^7^Department of Family and Consumer Studies, University of Utah, 225 S. 1400 E. Alfred Emery BLDG 228, Salt Lake City, UT 84112, USA; ^8^Department of Epidemiology, Rutgers University and Cancer Institute of New Jersey, 195 Little Albany Street, New Brunswick, NJ 089036-2681, USA; ^9^Department of Internal Medicine and University of New Mexico Cancer Center, University of New Mexico, 1 University Boulevard NE, Albuquerque, NM 87131, USA

## Abstract

*Background.* Population-based studies are needed to estimate the prevalence of underweight or overweight/obese childhood cancer survivors. *Procedure.* Adult survivors (diagnosed ≤20 years) were identified from the linked Utah Cancer Registry and Utah Population Database. We included survivors currently aged ≥20 years and ≥5 years from diagnosis (*N* = 1060), and a comparison cohort selected on birth year and sex (*N* = 5410). BMI was calculated from driver license data available from 2000 to 2010. Multivariable generalized linear regression models were used to calculate prevalence relative risks (RR) and 95% confidence intervals (95% CI) of BMI outcomes for survivors and the comparison cohort. *Results.* Average time since diagnosis was 18.5 years (SD = 7.8), and mean age at BMI for both groups was 30.5 (survivors SD = 7.7, comparison SD = 8.0). Considering all diagnoses, survivors were not at higher risk for being underweight or overweight/obese than the comparison. Male central nervous system tumor survivors were overweight (RR = 1.12, 95% CI 1.01–1.23) more often than the comparison. Female survivors, who were diagnosed at age 10 and under, had a 10% higher risk of being obese than survivors diagnosed at ages 16–20 (*P* < 0.05). *Conclusion.* While certain groups of childhood cancer survivors are at risk for being overweight/obese, in general they do not differ from population estimates.

## 1. Introduction 

As of 2005, there were over 328,000 childhood cancer survivors in the USA, a number that will continue to grow with emerging treatment procedures [1]. Unfortunately, survival from childhood cancer is often accompanied by an increased risk for adverse late effects from treatment [2–4], including cardiovascular risk [5, 6], insulin resistance [7], and neurologic, musculoskeletal, and pulmonary complications [8]. Furthermore, adult survivors of childhood cancer may be particularly prone to weight-related problems as approximately half report low levels of physical activity [9, 10]. In the general population, a high body mass index (BMI) in the overweight or obese range is associated with an increased risk for chronic health conditions including hypertension [11], diabetes [12], cancer [13], and cardiovascular disease [5, 14]. Late effects from treatment and low levels of physical activity may compound the risk of additional weight-related problems among survivors with abnormal BMIs.

There is a considerable body of evidence underscoring the impact of early life exposures, such as a pediatric cancer diagnosis, on health throughout the lifespan [4]. To date, most USA studies describing childhood cancer survivors' BMI have focused on samples of survivors diagnosed from 1970 to 1986 in the Childhood Cancer Survivor Study [15–19]. As many of the treatment protocols have evolved since that time, studies that include survivors diagnosed more recently are needed. Additionally, much of the research on childhood cancer survivors' BMI has emerged from clinical samples. With the high national prevalence of overweight and obesity [20] and with weight-related health problems emerging at younger ages [21], population-based studies can provide important context for determining policy and allocating resources to improve cancer survivors' long-term health.

Certain groups of childhood cancer survivors appear to face a higher risk of being overweight or obese, including survivors of acute lymphoblastic leukemia [22–24] and other leukemias [15] as do patients who are diagnosed at a young age [17–19, 25], female [18, 26], and recipients of cranial radiation [16, 18, 19]. Conversely, other childhood cancer survivors, such as those surviving Hodgkin disease and Wilms tumor, may instead be at risk for being underweight as adults [15]. For survivors of central nervous system tumors, the literature is mixed with some studies reporting elevated risk for being overweight or obese [25] as well as underweight [27], and others suggesting the weight distribution among these survivors is similar to that of the general population [28].

We conducted a population-based evaluation of BMI outcomes among adult survivors of childhood cancer. We queried a cohort of childhood cancer survivors diagnosed from 1973 to 2005 from the Utah Cancer Registry (UCR) and a comparison cohort sample from the Utah Population Database (UPDB) and Utah birth certificates. We hypothesized that childhood cancer survivors would be more likely to be overweight or obese in adulthood than the comparison cohort and that groups at highest risk of obesity would include leukemia survivors, female survivors, and those who received radiation therapy.

## 2. Methods

### 2.1. Data Resources

The UPDB is a University of Utah resource that contains over seven million individual records from statewide datasets [29, 30]. The UPDB includes all driver license records (as well as identification cards for nondrivers), which we used to ascertain self-reported height and weight. As over 80% of adults in the USA aged 20–70 have a driver license [31], using these data to ascertain BMI provides a high level of coverage typically unavailable through surveys, which often have lower response rates [32]. To validate the use of driver license data for BMI estimates, the UPDB has compared age- and sex-specific mean BMI values with two data sources, the 2000 Utah Behavioral Risk Factor Surveillance Survey (BRFSS) and 155 individuals with clinical measures of height and weight. BRFSS is used to assess BMI and obesity trends in the US (http://www.cdc.gov/brfss/), making it an appropriate data source to validate Utah driver license data. BRFSS mean BMI values were only 1% and 3% higher for males and females, respectively, in relation to driver license estimates. There was a high correlation between clinical and self-reported driver license height and weight (*r* = 0.85).

Cancer data were provided by the UCR, which has been a part of the Surveillance, Epidemiology, and End Results (SEER) Program of the National Cancer Institute since 1973. The UCR records are linked to the UPDB, with over 97% of individuals with cancer linked to one or more records in the UPDB [33]. All study protocols and procedures were approved by the University of Utah Institutional Review Board and the Utah Resource for Genetic and Epidemiologic Research.

### 2.2. Subject Sampling and Eligibility

A cohort of childhood cancer survivors was identified from the linked UCR and UPDB. A noncancer comparison cohort, with similar distribution of birth year and sex, was sampled from Utah birth certificates through the UPDB.

**Figure 1:**
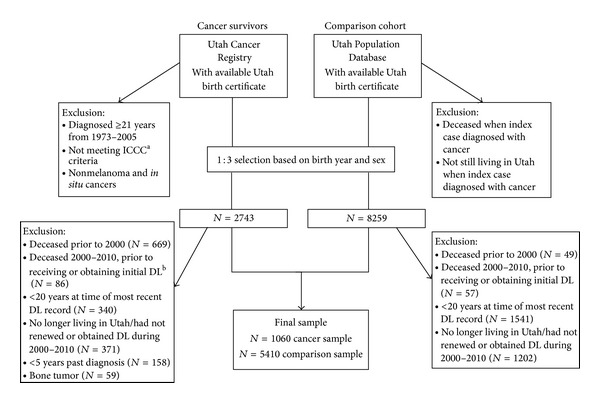
Sample exclusion criteria. ^a^International Classification of Childhood Cancers, ^b^Driver License.

*Childhood Cancer Cases.* The UCR was queried for all childhood cancer cases with a Utah birth certificate who were diagnosed in Utah before age 21 from 1973 to 2005. Eligible cases were diagnosed with a cancer that met the International Classification of Childhood Cancer (ICCC) criteria. The ICCC is the standard classification system for childhood cancers. It is based on tumor morphology and primary cancer site with an emphasis on morphology rather than cancer site as for adults [34]. Nonmelanoma skin cancers and cancers *in situ *were excluded. Ten cases were excluded due to lack of information on their diagnosis. A total of *N* = 2743 unique individuals were identified (Figure 1). 

*Comparison Cohort.* Noncancer participants were randomly selected from Utah birth certificates, which were accessed through the UPDB. The comparison sample was frequency matched on birth year and sex using a three to one ratio of comparison sample to cancer cases. A total of *N* = 8259 unique individuals were identified (Figure 1). 

*Eligibility Criteria for Cancer Survivors and the Comparison Cohort.* We limited our sample to individuals who survived to at least age 20 at the time of their most recent driver license record, because 20 is the minimum age for adult BMI calculations according to the National Heart, Lung, and Blood Institute (NHLBI) [35]. Other eligibility criteria included Utah driver license renewal during 2000–2010. Individuals in Utah are required by law to renew their driver license every five years and in person every ten years. Therefore, this ten-year date range captures at least one driver license renewal where driver license height and weight were updated. We excluded those who were either no longer living in Utah or had not renewed or obtained their initial driver license from 2000 to 2010. The survivor sample was also limited to those ≥5 years from diagnosis to ensure that a majority had completed their cancer therapy. Additionally, we excluded *N* = 59 bone cancer patients as we lacked information on amputations or limb-sparing therapy which can potentially affect weight and height. A total of *N* = 1060 survivors and *N* = 5410 in the comparison cohort were available for analysis. 

### 2.3. Demographic Measures

Sex and race/ethnicity were obtained from UPDB records. Age at BMI was calculated using the date seen in person for driver license renewal and date of birth.

### 2.4. Cancer-Related Measures

For cancer cases, the UCR provided data on diagnosis, date of diagnosis, age at diagnosis, receipt of surgery, chemotherapy and/or radiation as part of their first course therapy, and whether the individual had more than one primary cancer diagnosis. Time since diagnosis was calculated using BMI date and date of cancer diagnosis. Cancer diagnoses included lymphomas, leukemias (grouped as “other leukemia” and “acute lymphoblastic leukemia” (ALL)), central nervous system neoplasms (CNS), epithelial cancers (malignancies such as thyroid cancers and melanomas), germ/gonadal cancers, sarcomas, renal tumors, neuroblastomas, and retinoblastomas. Cancer treatment was categorized as eight mutually exclusive groups: surgery only, chemotherapy only, radiation only, chemotherapy/radiation, chemotherapy/surgery, radiation/surgery, and chemotherapy/radiation/ surgery, and not documented/no treatment. Second primary cancers were also tabulated (yes/no).

### 2.5. BMI Outcomes

The primary outcome of interest was BMI. Using the height and weight that were self-reported at the most recent driver license renewal, BMI was calculated as weight in kg/height in m^2^. We classified BMI according to the NHLBI standards: underweight (<18.5 kg/m^2^), normal (18.5–24.9 kg/m^2^), overweight (25–29.9 kg/m^2^), and obese (≥30 kg/m^2^) [36]. For our main analyses, abnormal BMIs were considered as underweight (BMI < 18.5) and overweight/obese (defined as BMI ≥ 25). These were evaluated as dichotomous outcomes with the other BMI categories as the referent (e.g., underweight versus normal-obese) to be comparable to other childhood cancer studies of BMI [15]. As a secondary analysis, obese (BMI ≥ 30) was analyzed as a dichotomous outcome compared to nonobese.

### 2.6. Statistical Analyses

All analyses were generated using statistical software, Stata 12.1. Descriptive statistics were calculated for demographic and cancer-related characteristics. Age at BMI, sex, and race/ethnicity distributions of the survivors and the comparison group were summarized in categories and tested using *χ* ^2^. Proportions were calculated for cancer-related factors (i.e., diagnosis, age at diagnosis, years since diagnosis, treatment, and second primary cancers).

Multivariable generalized linear regression models with robust standard errors were used to calculate prevalence relative risks (RR) and 95% confidence intervals (95% CI) both for survivors compared to the comparison cohort and for analyses limited to survivors only. A Gaussian family with identity link was used due to problems with model convergence using a binomial distribution. As BMI differs by sex [37], all analyses were run separately by sex. All models included an interaction term for continuous birth year and categorical age at BMI and were controlled for year at BMI measurement. We first estimated models to compare the outcomes of underweight and overweight/obese for the full cancer sample to the comparison sample. Then, to examine differences by diagnosis, we estimated regressions only among the top five most common cancer groups (lymphoma, epithelial, ALL, CNS, and germ cell) due to sample size limitations.

In our last set of models, we evaluated predictors of being underweight or overweight/obese among cancer survivors. Predictors of interest included age at diagnosis, race/ethnicity, and treatment type, as these factors have been associated with abnormal BMI in other childhood cancer studies [15]. As a secondary analysis, we examined obese (BMI ≥ 30) as a separate outcome for the survivor-only models. Also, as we were interested in understanding whether survivors with more recent diagnoses might have differences in BMI, these models were reestimated for those diagnosed 1990 and after. As the majority of our variables of interest were ascertained from birth and cancer registry records, missing data were minimal (less than 10% for most), so no analytics were used to address the potential bias due to missing data.

## 3. Results 

Age, sex, and race/ethnicity did not differ significantly between survivors and the comparison cohort in Table 1. Average time since diagnosis was 18.5 (SD = 7.8), and mean age at BMI was 30.5 for both the survivors (SD = 7.7) and comparison group (SD = 8.0). Surgery only (23.1%) and chemotherapy only (19.5%) were the most common treatment groups. In Table 2, the most common cancers among female survivors were epithelial (26.1%) and lymphoma (17.4%), and for males, lymphoma (23.8%) and CNS tumors (16.3%). There were no differences between the combined survivor group and the comparison group in distributions of BMI categories (underweight, normal, overweight, and obese).

### 3.1. BMI among Cancer Survivors and Comparison Cohort for the Five Most Common Cancer Diagnoses

We estimated models comparing survivors to the comparison cohort for the overall cancer sample and by cancer diagnosis in Table 3. For the overall survivor group, there were no differences for either female or male survivors versus the comparison cohort in their risk of being underweight or overweight/obese. However, when examined by cancer diagnosis, female epithelial survivors were less likely to be overweight or obese (RR = 0.89, 95% CI 0.82–0.96) than the comparison. Among males, CNS tumor survivors had a slightly higher risk of being overweight or obese (RR = 1.12, 95% CI 1.01–1.23) than the comparison.

### 3.2. BMI Outcomes among Cancer Survivors by Age at Diagnosis, Race/Ethnicity, and Treatment Therapy

We then estimated regression models for survivors only to evaluate the impact of age at diagnosis, race/ethnicity, and cancer therapy on risk of being underweight or overweight/obese in separate models by sex in Table 4. In our main models we found that, for female survivors, cancer therapy was not significantly associated with being underweight or overweight/obese. Younger diagnosis age was marginally significant for being overweight/obese for females aged 5–10 years at diagnosis (RR = 1.14, 95% CI 1.00–1.31) compared to ages 16–20, and the test for trend across age groups was significant at *P* = 0.03. Non-Hispanic White female survivors tended to be underweight (RR = 1.09, 95% CI 1.04–1.15) more often than survivors of Other races. No factors were statistically significant in the male survivors' models.

As a secondary analysis, we also examined the risk of being obese among survivors. Female survivors diagnosed aged ≤4 years (RR = 1.12, 95% CI 1.03–1.22) and 5–10 years (RR = 1.10, 95% CI 1.01–1.21) were at higher risk for being obese when compared to those diagnosed aged 16–20 years (*P*  value test for trend *P* = 0.004). Non-Hispanic White female survivors were more likely to be obese than female survivors of other races (RR = 1.11, 95% CI 1.04–1.19). Female survivors with chemotherapy and surgery had a lower risk of obesity (RR = 0.90, 95% CI 0.84–0.96) compared to patients receiving only surgery. Finally, when we restricted our analyses to survivors diagnosed after 1990, no differences emerged in the impact of age, race/ethnicity, and cancer therapy on risk of being underweight or overweight/obese for either female or male survivors.

## 4. Discussion 

This study is one of the first population-based evaluations of prevalence of underweight and overweight/obese adult survivors of childhood cancer. Our findings expand on earlier studies by utilizing a large state-level sample of survivors diagnosed from 1973 to 2005. We found that, among adult survivors of childhood cancer in Utah, 36% of females and 61% of males had BMIs that categorized them as overweight or obese, although these prevalences were similar to an age- and sex-matched comparison cohort from the general population. Other studies have reported that survivors' prevalence of overweight is not higher than population-based controls [24]. In addition, although treatment protocols have changed during the past decades, we found no differences in the impact of cancer therapy when examining survivors diagnosed after 1990.

Few differences in BMI were found by cancer diagnosis. Female survivors of epithelial cancers were less likely to be overweight or obese in reference to the comparison cohort. Only male CNS tumor survivors were at an elevated risk of being overweight or obese, similar to prior research [25]. This finding is not surprising as the treatment for brain tumors often includes cranial radiation. As a result, hypothalamic function can be affected, which may potentially predispose these survivors to weight problems at a higher rate than other childhood cancer survivors [38]. Conversely, while an earlier report from the Childhood Cancer Survivor Study found that leukemia survivors were more often obese compared to population norms [15] neither ALL nor other leukemia survivors in our sample showed differences.

Although our findings suggest that most childhood cancer survivors are not at an elevated risk for abnormal BMI, we did see that certain groups of survivors face a higher risk of obesity. Specifically, female survivors diagnosed between the ages of 0 and 10 years had a modest increased risk of obesity when compared to those diagnosed at older ages, concurrent with findings from prior studies [17–19, 25]. A similarly elevated risk was found for Non-Hispanic White female survivors compared to survivors of Other races. However, due to the small proportion of Other race/ethnicity participants, we are limited in understanding the implications of this finding. In the future, particular focus should be given to developing population-based studies that include more racially/ethnically diverse survivor populations as, in the general population, they tend to have higher BMIs than Non-Hispanic Whites [23]. In addition, women historically have had higher rates of overweight/obesity in the USA, yet in recent years (i.e., 2009-2010) this difference subsided [20]. In Utah, substantially more men are overweight or obese (70%) than women (52%) [39]. Similarly, we found that males in both the survivor and comparison sample had higher proportions of overweight/obese than females.

Our study has limitations. First, we did not have detailed information on cancer therapy (e.g., amputations, chemotherapy type, and duration of therapy) limiting our ability to identify the effect of specific therapies on BMI. Second, driver license data is self-reported and may not be as accurate as clinical methods of measuring BMI. Third, more women underreport their weight than men [40]. Fourth, although self-reported data tend to underestimate BMI values in relation to clinical data, as discussed earlier, the UPDB's validation of Utah driver license data to BRFSS and a clinical sample found BMI to be comparable across the data sources. Fifth, the matching of survivors to the comparison cohort was done early in the selection process. The data were selected and matched using several different statewide data sources, and due to practical limitations, it was not possible to impose all of the exclusions at the initiation of the study. However, despite this limitation, the two samples are very similar on age and sex.

Though still high, Utah has a lower prevalence of overweight and obesity than the US population [41]. We evaluated BMI at only one time point; thus, our results could be affected by survivor bias. While we have no reason to believe survivors would report their weight differently than the comparison, some survivors have poorer functional health [42]. These survivors may have more difficulty with day-to-day activities such as driving and, therefore, may be less likely to obtain driver licenses. Thus, our results may not reflect survivors with poorer health outcomes. In addition, longitudinal studies are needed to provide detailed information on the long-term risks for childhood cancer survivors. Although we only had cross-sectional information on BMI, our study reports on survivors diagnosed in recent years. As such, our study expands on earlier cross-sectional studies as more recent changes in treatment and long-term management of childhood cancer patients are likely to be reflected in our results. Finally, some studies have used clinic-based, rather than population-based, ascertainment of cases and used different comparison groups. Thus, our results may be more generalizable than these studies as our cases and comparison group were drawn from the same population.

## 5. Conclusions

In light of these data, childhood cancer patients and their families can be reassured that cancer therapy is unlikely to have a large impact on adult BMI. However, childhood cancer survivors remain at risk for developing late effects that could be exacerbated by an abnormal BMI, and 36% of female and 61% of male survivors in Utah are overweight or obese. Survivors have higher risk of developing diabetes, high blood pressure and cholesterol, osteonecrosis, cardiovascular complications, and stroke than the general population [43, 44]. Moreover, childhood cancer survivors often do not achieve the recommended guidelines for physical activity [45] and sustaining a healthy diet [46]. Given their susceptibility to certain health problems, the high prevalence of overweight and obesity that we observed among survivors of childhood cancer, although similar to the general population, is of concern. Diet, nutrition, and physical activity guidelines for cancer survivors have been developed by the American Cancer Society [47], yet most exercise and diet interventions for childhood cancer survivors have had a modest impact on behavior [48, 49]. Thus, childhood cancer survivors can benefit from access to resources to help them maintain a healthy weight and to minimize their risk for late effects. Additional research to identify effective strategies for promoting healthy body weight to minimize late effects risk for childhood cancer survivors is needed [15, 49].

## Figures and Tables

**Table 1:** Demographics and cancer-related factors for survivors and the comparison cohort.

	Cancer survivors		Comparison cohort		
	(*N* = 1060)		(*N* = 5410)		*P* value
	*N*	%	*N*	%
Age at body mass index					
18–29	565	53.3	2953	54.6	0.62
30–39	351	33.1	1709	31.6	
≥40	144	13.6	748	13.8	
Sex					
Female	518	48.8	2665	49.2	0.82
Male	542	51.2	2745	50.7	
Race/ethnicity					
White, Non-Hispanic	1036	97.6	5261	97.1	0.37
Other	24	2.4	149	2.8	
Birth year					
1952–1960	59	5.6	320	5.9	0.40
1961–1970	181	17.1	913	16.9	
1971–1980	468	44.1	2253	41.7	
1981–1990	352	33.2	1924	35.6	
Diagnosis age (years)					
≤4	214	20.9	NA		NA
5–10	178	16.8			
11–15	237	22.4			
16–20	431	40.7			
Years since diagnosis					
5–10	191	18.0	NA		NA
11–20	414	39.1			
21–30	363	32.6			
31–38	92	8.3			
Diagnosis year					
1973–1979	161	15.2	NA		NA
1980–1989	402	37.9			
1990–1999	376	35.5			
2000–2005	121	11.4			
Treatment					
None/not documented	170	16.0	NA		NA
Chemotherapy	207	19.5			
Radiation	119	11.2			
Surgery	245	23.1			
Chemotherapy + radiation	149	14.1			
Chemotherapy + surgery	73	6.9			
Radiation + surgery	52	4.9			
Chemotherapy + radiation + surgery	45	4.3			
Second primary cancers					
No	1052	99.2	NA		NA
Yes	8	0.8			

**Table 2:** Body Mass Index proportions for survivors by cancer type and comparison cohort.

			Body Mass Index								
	Total		Underweight		Normal		Overweight		Obese		
			BMI < 18.5		BMI 18.5–24.9		BMI 25–29.9		BMI ≥ 30		*P* value
	*N*	%	*N*	%	*N*	%	*N*	%	*N*	%	
Female											
Comparison sample	n/a		123	4.6	1551	58.2	598	22.4	393	14.8	0.06^b^
All cancers	n/a		33	6.4	296	57.1	130	25.1	59	11.4	
Diagnosis groups											
Lymphoma	90	17.4	8	8.9	53	58.9	22	24.2	7	7.0	
Epithelial	135	26.1	7	5.2	87	64.4	25	18.5	16	11.9	
ALL^a^	82	15.9	6	7.3	43	52.4	22	27.1	11	13.4	
Central nervous system	67	13.0	4	6.0	36	53.7	17	25.4	10	14.9	
Germ	34	6.6	0	0.0	22	61.8	11	33.3	2	5.9	
Sarcoma	41	7.9	5	12.2	19	46.3	13	31.7	4	9.7	
Renal	25	4.8	1	4.0	13	52.0	8	32.0	3	12.0	
Neuroblastoma	16	3.1	0	0.0	9	56.3	4	25.0	3	18.8	
Other leukemia	14	2.7	1	7.1	8	57.1	3	21.4	2	14.3	
Retinoblastoma	13	2.5	1	7.7	7	53.9	4	30.8	1	7.7	
Male											
Comparison sample	n/a		31	1.1	1102	40.2	1083	39.5	529	19.3	0.13^c^
All cancers	n/a		11	2.0	198	36.6	216	39.9	117	21.6	
Diagnosis groups											
Lymphoma	129	23.8	1	0.8	46	35.6	51	39.5	31	24.0	
Epithelial	64	11.8	1	1.6	22	34.4	26	40.6	15	23.4	
ALL^a^	83	15.3	2	2.4	33	39.8	32	38.6	32	19.3	
Central nervous system	88	16.3	0	0.0	28	31.8	35	39.8	25	28.4	
Germ	83	15.3	4	4.8	35	42.2	29	34.9	15	18.1	
Sarcoma	36	6.7	0	0.0	15	41.7	14	38.9	7	19.4	
Renal	25	4.6	0	0.0	5	20.0	16	64.0	4	16.0	
Neuroblastoma	18	3.3	1	5.6	9	50.0	6	33.3	2	11.1	
Other leukemia	11	2.0	1	9.1	4	36.4	6	54.6	0	0.0	
Retinoblastoma	4	0.7	1	25.0	0	0.0	1	25.0	2	50.0	

^a^Acute lymphoblastic leukemia.

^b^Comparing full female comparison cohort to full female cancer group.

^c^Comparing full male comparison cohort to full male cancer group.

**Table 3:** Relative risks (RR) and 95% confidence intervals (95% CI) of BMI outcomes for all cancers and the five most common cancers versus comparison cohort^a^.

	Underweight			Overweight/obese		
	(BMI < 18.5)^a,b^			(BMI ≥ 25)^a,c^		
	RR	95% CI	*P*-value	RR	95% CI	*P*-value
Female						
Comparison cohort (ref)	1			1		
All cancers	1.02	1.00–1.04	0.10	0.99	0.94–1.03	0.58
Top five cancers						
Lymphoma	1.05	0.98–1.11	0.14	0.94	0.86–1.04	0.24
Epithelial	1.01	0.98–1.05	0.44	**0.89**	**0.82–0.96**	**0.004**
Acute lymphoblastic leukemia^b^	1.02	0.96–1.08	0.47	1.07	0.96–1.19	0.22
Central nervous system	1.01	0.96–1.07	0.69	1.03	0.92–1.16	0.58
Germ	n/a^d^			0.97	0.82–1.14	0.70
Male						
Comparison cohort (ref)	1			1		
All cancers	1.01	1.00–1.02	0.16	1.02	0.98–1.07	0.28
Top five cancers						
Lymphoma	1.00	0.98–1.01	0.74	1.03	0.95–1.11	0.52
Epithelial	1.01	0.98–1.04	0.61	1.00	0.89–1.13	0.99
Acute lymphoblastic leukemia	1.01	0.98–1.04	0.58	1.03	0.93–1.13	0.54
Central nervous system	n/a^d^			**1.12**	**1.01–1.23**	**0.03**
Germ	1.04	0.99–1.09	0.10	0.91	0.82–1.00	0.06

^a^Models included both main effects and an interaction term for continuous birth year and categorical age at BMI and were adjusted for year at BMI measurement. For females, the full cancer model includes *N* = 518 cancers and *N* = 2665 in the comparison. For males, the full cancer model includes *N* = 542 cancers and *N* = 2745 in the comparison.

^b^Underweight versus Normal-Obese.

^c^Overweight/Obese versus Underweight-Normal.

^d^Female germ cell and male central nervous system not estimated as no cases were underweight in these cancer groups.

Bold indicates significant at *α* < 0.05.

**Table 4:** Relative Risks (RR) and 95% Confidence Intervals (95% CI) of BMI outcomes for survivors by age at diagnosis, race, and treatment therapy.

	Main analyses^a,c^						Secondary analysis^b,c^		
	Underweight (BMI < 18.5)^3^			Overweight/obese (BMI ≥ 25)^3^			Obese (BMI ≥ 30)^3^		
	RR	95% CI	*P*-value	RR	95% CI	*P*-value	RR	95% CI	*P*-value
Female (*N* = 518)									
Diagnosis age									
16–20 (ref)	1			1			1		
11–15	0.98	0.93–1.04	0.56	1.04	0.94–1.16	0.42	1.05	0.98–1.13	0.13
5–10	0.95	0.89–1.02	0.16	**1.14**	**1.00–1.31**	**0.05**	**1.10**	**1.01–1.21**	**0.04**
≤4	0.97	0.90–1.04	0.37	1.14	0.99–1.31	0.06	**1.12**	**1.03–1.22**	**0.007**
Race/ethnicity									
Other (ref)	1			1			1		
White, Non-Hispanic	**1.09**	**1.04–1.15**	**0.001**	0.95	0.75–1.21	0.67	**1.11**	**1.04–1.19**	**0.002**
Cancer therapy									
Surgery (ref)	1			1			1		
None or not documented	1.00	0.95–1.06	0.89	1.08	0.93–1.25	0.32	0.93	0.85–1.02	0.14
Chemotherapy	1.03	0.96–1.11	0.35	0.98	0.86–1.11	0.74	0.96	0.89–1.04	0.35
Radiation	1.06	0.97–1.16	0.17	0.99	0.84–1.15	0.88	0.97	0.88–1.08	0.62
Chemotherapy + radiation	1.01	0.96–1.07	0.67	1.15	0.99–1.34	0.06	0.97	0.88–1.07	0.62
Chemotherapy + surgery	1.08	0.94–1.24	0.26	1.05	0.87–1.27	0.61	**0.90**	**0.84–0.96**	**0.001**
Radiation + surgery	1.01	0.90–1.13	0.87	1.17	0.95–1.42	0.13	1.18	0.98–1.41	0.08
Chemotherapy + radiation + surgery	1.11	0.95–1.30	0.19	0.95	0.78–1.16	0.61	0.98	0.88–1.09	0.68

Male (*N* = 542)									
Diagnosis age									
16–20 (ref)	1			1			1		
11–15	1.00	0.97–1.03	0.92	1.07	0.94–1.21	0.30	1.01	0.90–1.12	0.91
5–10	0.98	0.95–1.02	0.43	1.00	0.88–1.15	0.95	0.95	0.85–1.06	0.33
≤4	1.01	0.97–1.05	0.56	1.11	0.96–1.27	0.15	0.96	0.85–1.08	0.49
Race/ethnicity									
Other (ref)	1			1			1		
White, Non-Hispanic	0.94	0.80–1.10	0.44	0.94	0.72–1.22	0.64	1.03	0.93–1.28	0.79
Cancer therapy									
Surgery (ref)	1			1			1		
Chemotherapy	0.99	0.95–1.04	0.78	0.99	0.88–1.13	0.93	1.03	0.92–1.15	0.63
Radiation	1.00	0.96–1.04	0.94	0.98	0.83–1.16	0.83	1.14	0.97–1.34	0.11
Not documented/no treatment	1.03	0.97–1.10	0.28	1.01	0.86–1.19	0.88	1.09	0.94–1.26	0.23
Chemotherapy + radiation	0.99	0.94–1.04	0.71	0.91	0.78–1.05	0.21	0.97	0.86–1.09	0.59
Chemotherapy + surgery	0.99	0.94–1.04	0.74	1.01	0.85–1.20	0.93	1.00	0.87–1.14	0.98
Radiation + surgery	0.97	0.93–1.00	0.09	0.98	0.78–1.21	0.83	1.01	0.84–1.22	0.92
Chemotherapy + radiation + surgery	0.96	0.93–1.00	0.05	1.06	0.87–1.29	0.57	1.05	0.89–1.24	0.55

^a^Main analyses include two models; the first model compared underweight to all other BMI categories. The second model compared overweight/obese as one category to all other BMI categories.

^b^Secondary analysis model compared obese to all other BMI categories.

^c^Models included both main effects and an interaction term for continuous birth year and categorical age at BMI and were adjusted for year at BMI measurement.

Bold indicates significant at *α* < 0.05.
